# Aging is associated with an insufficient early inflammatory response of lung endothelial cells in SARS-CoV-2 infection

**DOI:** 10.3389/fimmu.2024.1397990

**Published:** 2024-06-07

**Authors:** Saravanan Subramaniam, Devin Kenney, Archana Jayaraman, Aoife Kateri O’Connell, Sarah Walachowski, Paige Montanaro, Christoph Reinhardt, Giuseppe Colucci, Nicholas A. Crossland, Florian Douam, Markus Bosmann

**Affiliations:** ^1^ Pulmonary Center, Department of Medicine, Boston University Chobanian and Avedisian School of Medicine, Boston, MA, United States; ^2^ National Emerging Infectious Diseases Laboratories (NEIDL), Boston University, Boston, MA, United States; ^3^ Department of Virology, Immunology and Microbiology, Boston University Chobanian and Avedisian School of Medicine, Boston, MA, United States; ^4^ Department of Pathology and Laboratory Medicine, Boston University Chobanian and Avedisian School of Medicine, Boston, MA, United States; ^5^ Center for Thrombosis and Hemostasis, University Medical Center of the Johannes Gutenberg-University, Mainz, Germany; ^6^ German Center for Cardiovascular Research (DZHK), Partner Site Rhine-Main, Mainz, Germany; ^7^ Outer Corelab, Viollier AG, Allschwil, Switzerland; ^8^ Department of Hematology, University of Basel, Basel, Switzerland

**Keywords:** host-pathogen interaction, pattern recognition receptors, cytokines, inflammation, thromboinflammation

## Abstract

Advanced age is associated with an increased susceptibility to Coronavirus Disease (COVID)-19 and more severe outcomes, although the underlying mechanisms are understudied. The lung endothelium is located next to infected epithelial cells and bystander inflammation may contribute to thromboinflammation and COVID-19-associated coagulopathy. Here, we investigated age-associated SARS-CoV-2 pathogenesis and endothelial inflammatory responses using humanized K18-hACE2 mice. Survival was reduced to 20% in aged mice (85–112 weeks) versus 50% in young mice (12–15 weeks) at 10 days post infection (dpi). Bulk RNA-sequencing of endothelial cells from mock and infected mice at 2dpi of both age groups (aged: 72–85 weeks; young: 15 weeks) showed substantially lower significant differentially regulated genes in infected aged mice than in young mice (712 versus 2294 genes). Viral recognition and anti-viral pathways such as RIG-I-like receptor signaling, NOD-like receptor signaling and interferon signaling were regulated in response to SARS-CoV-2. Young mice showed several fold higher interferon responses (*Ifitm3*, *Ifit1*, *Isg15, Stat1*) and interferon-induced chemokines (*Cxcl10* and *Cxcl11*) than aged mice. Endothelial cells from infected young mice displayed elevated expression of chemokines (*Cxcl9*, *Ccl2*) and leukocyte adhesion markers (*Icam1*) underscoring that inflammation of lung endothelium during infection could facilitate leukocyte adhesion and thromboinflammation. TREM1 and acute phase response signaling were particularly prominent in endothelial cells from infected young mice. Immunohistochemistry was unable to detect viral protein in pulmonary endothelium. In conclusion, our data demonstrate that the early host response of the endothelium to SARS-CoV-2 infection declines with aging, which could be a potential contributor to disease severity.

## 1 Introduction

Coronavirus disease 2019 (COVID-19) is caused by the severe acute respiratory syndrome coronavirus 2 (SARS-CoV-2) with phenotypes ranging from asymptomatic to severe complications resulting in multiple organ failure and death (1, 2). With the widespread vaccination campaigns and herd immunity, the COVID-19 pandemic is approaching an endemic phase but remains a persistent threat as the viral genome continues to mutate. Moreover, COVID-19 has a disproportionate morbidity depending on the patient’s age. The Centers for Disease Control and Prevention (CDC) statistics revealed that compared with ages 18–29 years, mortality is 3.5-fold higher in ages 30–39 years, and 350-fold higher in individuals aged >85 years, demonstrating a substantial age-dependent increase in disease severity (3). Comorbidities, such as cardiovascular disease and diabetes mellitus in older adults, likely contribute to severe outcomes, but the pathogenic mechanisms of severe COVID-19 in elderly patients remain incompletely understood (4).

Patients with dysfunctional endothelial cells (ECs), which line the luminal side of the blood vessels, due to unresolved inflammation and stress have a higher risk of thrombosis and mortality from organ failure (5). Endothelium and blood vessels are vulnerable to COVID-19-induced tissue injury (6) and are prone to develop an inflammatory milieu associated with spontaneous thrombosis in their arterial and venous beds, which can result in deadly conditions such as pulmonary embolism, deep vein thrombosis, arterial thrombosis, and organ failure (1, 7, 8). SARS-CoV-2 does not appear to have intrinsic procoagulant effects itself; rather, coagulopathy may arise from the profound COVID-19-associated inflammatory response and endothelial activation/damage (9). COVID-19 autopsy reports claim pulmonary endothelial viral inclusions and apoptosis, increased angiogenesis, and increased capillary microthrombi (10, 11). However, investigators have inaccurately reported subcellular structures as coronavirus particles and thus these observations remain debatable (12). In humans, there is little convincing evidence for viral dissemination and replication outside of the respiratory tract. The extrapulmonary disease is likely attributable to the systemic inflammation, not viral dissemination to other organs. Furthermore, COVID-19 convalescent subjects experience ongoing cardiovascular issues such as coagulopathy, bleeding disorders and thromboinflammation (13, 14).

Approximately 30–50% of COVID-19 patients treated in emergency care units experienced arterial and venous thromboembolism despite the regular use of thromboprophylaxis, suggesting that advanced treatment for endothelial impairment could be beneficial to prevent thrombosis (15–19). However, it remains unclear whether the contribution of ECs to hypercoagulation and hyperinflammation is due to endothelial injury or dysfunction, direct SARS-CoV-2 infection, or mediated indirectly through bystander inflammation in vicinity to infected lung epithelial cells.

In our present study, unbiased whole-transcriptome sequencing of isolated lung ECs from aged humanized transgenic ACE2 mice showed less induction of adhesive markers (e.g., *Icam1*) and inflammatory chemokines (e.g., *Ccl2*), and impaired interferon (IFN) response, and TREM1-signaling compared to young mice at 2 days post infection (2dpi). Immunohistochemistry of lungs revealed that the ECs were lacking SARS-CoV-2 viral particle (N protein). Thus, the responses seen in the pulmonary ECs in humanized transgenic ACE2 mice may be indirect effects mediated by local epithelial infection in lung. Overall, our findings support that the age-associated pulmonary endothelial dysfunction is likely shaped by a diminished immune response, which may contribute to disease severity.

## 2 Methods

### 2.1 Mice

Heterozygous transgenic humanized ACE2 (K18-hACE2) mice (strain: 2B6.Cg-Tg(K18-ACE2)2Prlmn/J) were obtained from the Jackson Laboratory (Bar Harbor, ME) and maintained in Tecniplast green line individually ventilated cages (Tecniplast, Buguggiate, Italy). Mice were maintained on a 12:12 light cycle at 30–70% humidity and provided *ad libitum* water and standard chow diets (LabDiet, St. Louis, MO, USA). Experimental procedures with animals were approved by the Boston University Biomedical Research, Institutional Biosafety Committee and Institutional Animal Care and Use Committee (IACUC).

### 2.2 SARS-CoV-2 propagation

The propagation of SARS-CoV-2 followed established procedures (20). The SARS-CoV-2 isolate 2019-nCoV/USA-WA1/2020 (NCBI accession number: MN985325; WA-1) was obtained from the CDC (Atlanta, GA, USA) and BEI Resources (Manassas, VA, USA). African green monkey kidney Vero E6 cells (ATCC^®^ CRL-1586™, American Type Culture Collection, Manassas, VA) were seeded at a concentration of 1x10^7^ cells in a T175 flask one day before virus generation. Then, the cells were infected with the virus diluted in 10 mL of Opti-MEM (ThermoFisher Scientific, Waltham, MA, USA) and incubated for 1 hour at 37°C for virus adsorption. Thereafter, 15 mL of DMEM containing 10% FBS and 1% penicillin/streptomycin was added, and the cells were incubated overnight. Subsequently, the media was removed, cells were rinsed with 1X PBS, pH 7.5 (ThermoFisher Scientific), and 25 mL of fresh DMEM containing 2% FBS was added. The cells were monitored for cytopathic effect, the media was harvested, filtered through a 0.22 μm filter, and concentrated using a sucrose gradient. The concentrated virus was suspended in sterile 1X PBS, pH 7.5, aliquoted, and stored at −80°C.

### 2.3 SARS-CoV-2 titration via plaque assay

Viral stock titration was evaluated using plaque assay. Vero E6 cells were seeded into a 12-well plate at a concentration of 2x10^5^ cells/well. The next day, the cells were exposed to 10-fold serially diluted viral stock and incubated for 1 hour at 37°C. Afterwards, 1 mL of overlay media (comprising 1.2% Avicel (DuPont, Wilmington, DE, USA; RC-581) in DMEM with 2% FBS and 1% Pen/Strep) was added per well. Three days later, the overlay media was discarded, and the cells were fixed with 10% neutral buffered formalin (ThermoFisher Scientific) for 1 hour at room temperature. Subsequently, the formalin was removed, and the cells were stained with 0.1% crystal violet (Sigma-Aldrich) in 10% ethanol/water for 30 min at room temperature. The stain was rinsed off, cells were washed with water, and plaque-forming units (PFU) were counted to determine viral titers.

### 2.4 SARS-CoV-2 infection of mice

Male and female K18-hACE2 transgenic mice (12–15 and 72–112 weeks of age) were intranasally inoculated with 1x10^4^ (survival) or 1x10^6^ (endpoint studies) PFU of SARS-CoV-2 in 50 μL of sterile 1X PBS or sham inoculated. Inoculations were performed under 1–3% isoflurane anesthesia. Survival was assessed up to 12dpi. Mice were euthanized with ketamine/xylazine at predetermined time points for sample collection or earlier if they met euthanasia criteria (defined by an IACUC-approved clinical scoring system).

### 2.5 Clinical monitoring

An IACUC-approved clinical scoring system was used to monitor disease progression and to establish humane endpoints of infected mice (21). The evaluated categories were body weight, general appearance, responsiveness, respiration, and neurological signs. Clinical signs and body temperature were recorded once daily for the full duration of the studies.

### 2.6 Lung dissociation and EC isolation

Microvascular lung ECs were isolated from K18-hACE2 mice infected with 1x10^6^ PFU of SARS-CoV-2 or mock-infected K18-hACE2 mice. Lungs were placed in DMEM containing 2% FBS prior to dissociation with a Miltenyi Biotec mouse lung dissociation kit (cat.# 130–095-927) following the manufacturer’s protocol. Tissues were minced using a Miltenyi GentleMACS, filtered through a 70-μm cell strainer, centrifuged at 300 *x g* at 4°C for 8 min, and cell pellets were suspended in MACS buffer prior to endothelial isolation.

ECs were isolated using a two-step sorting method. First, cells were negatively selected with a CD45 Microbead mouse kit (Miltenyi Biotec; cat.# 130–052-301) per manufacturer’s protocol. The collected negative fraction was washed thrice with MACS buffer prior to a positive selection for lung ECs using a CD31 Microbead mouse kit (cat.# 130–097-418) following the manufacturer’s protocol. The positive fraction was washed thrice with MACS buffer then resuspended in 500 μL of MACS buffer. All sorting protocols were performed on an AutoMACS Pro Cell separator using manufacturer’s recommended settings. After final isolation and washing, 50 μL of each sample was checked for purity by flow cytometry. The remaining 450 μL was centrifuged at 300 *x g* at 4°C for 8 min and the cell pellets were lysed in 600 μL QIAGEN RLT buffer containing beta-mercaptoethanol for RNA isolation (QIAGEN, Venlo, Netherlands).

### 2.7 Flow cytometry

Cell suspensions (50 µL) from the positive or the negative fraction were stained using surface staining protocol as previously described (22) with the PE-conjugated CD31 (PECAM-1) monoclonal antibody (clone 390, 1:100) and isotype control (clone eBR2a) (eBioscience, USA). Samples were fixed with 4% paraformaldehyde for 1 hour then washed twice with 1X PBS. Sample acquisition was performed on a LSRII instrument (BD Biosciences, USA), and data were analyzed using FlowJo v10.10.0 (FlowJo, Ashland, OR, USA).

### 2.8 Histology and immunohistochemistry

The lungs were prepared as described before (21). Immunohistochemistry was performed using a Ventana Discovery Ultra (Roche, Basel, Switzerland). The following monoclonal antibodies were used: mouse SARS-CoV-2 Nucleocapsid protein (clone:1C7C7; Cell Signaling Technology, Danvers, MA, USA); rabbit mouse on mouse linking antibody (clone M204–3; Abcam, Waltham, MA, USA), HRP anti-rabbit IgG polymer (Vector Labs, Neward, CA, USA), developed with 3,3’-Diaminobenzidine, and counter stained with hematoxylin. Slides were imaged using a Vectra Polaris whole slide scanner (Akoya Biosciences, Marlborough, MA, USA) and analyzed utilizing the HALO™ image analysis platform (Indica labs, Albuquerque, NM, USA).

### 2.9 RNA-seq

RNA was extracted using QIAGEN RNAeasy Plus micro kit. Ultra-low input RNA-seq was performed by Genewiz (Azenta US, Inc., NJ, USA). The FASTQ (150bp; paired-end) files from Genewiz were aligned to a custom combined reference (FASTQ and GTF) of mouse (GRCm39, Gencode v27) and SARS-CoV-2 (isolate Wuhan-Hu-1; NCBI accession ID: NC_045512.2) genomes using STAR aligner (v2.7.9a). The aligned reads were indexed and sorted using samtools (v1.10) and quantified using featureCounts from the subread (v1.6.2) (23) package. Further analyses were performed in RStudioServer/R (v4.1.1) framework with the DESeq2 (v1.34.0) (24) package. The principal components were visualized using pcaExplorer (v2.20.1) (25) after regularized log transformation and variance stabilizing transformation (vst) of counts. Differential expression (DE) analysis was based on Wald’s test with multiple test adjustment using Benjamini-Hochberg (BH) method in DESeq2 and shrinkage of log2-fold changes using the apeglm (v1.16.0) (26) package. The mouse DE genes (DEGs) were annotated from Ensembl (release 104) through the biomaRt (v2.50.1) (27) package; for the SARS-CoV-2 DEGs, gene symbols from the quantification step were retained. Statistical significance threshold was set at adjusted P<0.05. Venn data were estimated from the VennDetail (v1.10.0) (28) package. Kyoto Encyclopedia of Genes and Genomes (KEGG) pathway enrichment for significant DEGs was performed using clusterProfiler (v4.2.1) (29) with BH multiple testing, and pathways with adjusted P<0.05 considered statistically significant. QIAGEN Ingenuity Pathway Analysis (IPA, QIAGEN Inc., https://digitalinsights.qiagen.com/IPA) (30) was also performed with pathway significance threshold set at P<0.05 based on right-tailed Fisher’s exact test, and the enrichment was restricted to ECs through IPA’s tissue and cell filtering option. Heatmaps were constructed using the vst counts for DEGs with baseMean>50 with the ComplexHeatmap (v2.10.0) (31) package and all other figures were generated using ggplot2 (v3.3.5) (32). The heatmap visualizing z-scores for IPA enrichment was constructed using GraphPad Prism (v9.5.1; GraphPad Software, San Diego, CA, USA). Weighted gene co-expression network analysis (WGCNA) was performed using the WGCNA package (v1.72–5) (33) to identify co-regulated genes. The vst counts data were used as input after filtering out outlier genes as determined by the goodSamplesGenes function of WGCNA. The scale free topology was analyzed at different powers ranging from 10 to 30 for signed networks. However, as none of the powers reached an R^2^ of 0.9, potentially due to biological heterogeneity between aged and young mice, we selected a soft-thresholding power of 18 as suggested by the WGCNA developers for signed networks for <20 samples (34). The network construction and module detection were performed using the blockwise approach with the parameters: maxBlockSize = 50000, TOMType = “signed”, power = 18, numericLabels = TRUE, randomSeed = 1234, saveTOMS = TRUE, minModuleSize = 30, mergeCutHeight = 0, pamRespectsDendro = FALSE (35). The correlation (Pearson) between module eigengenes and experimental groups and its statistical significance (Student asymptotic p-value) were calculated through WGCNA. A module eigengene represents the expression profiles of genes in a module (33). The experimental groups were coded in a binary format (0/1) through WGCNA’s binarizeCategoricalColumns function and included the groups aged mock, aged 2dpi, young mock, young 2dpi. In addition, correlation between eigengenes and infection status (binarized format: infected = 1, uninfected = 0) and age (aged = 1, young = 0) were also assessed. Gene ontology biological process (GO BP) enrichment of modules of biological relevance was performed using clusterProfiler with BH multiple testing, and processes with adjusted P<0.05 considered statistically significant. Genes that showed significant positive correlation with the module (correlation >0, correlation P<0.05) were used for the enrichment analysis. Correlation heatmap was visualized using pheatmap (v1.0.12) and module eigengene heatmaps were constructed using ComplexHeatmap. All other plots were generated using ggplot2. WGCNA heatmap and boxplot were based on code from (36).

### 2.10 Statistical analysis

Statistical analysis was performed with Prism v8 software (GraphPad). Sample sizes and number of technical and biological replicates are included in the figure legends. Data in the bar graphs represent mean ± standard error of the mean (s.e.m.). Comparison of two groups was performed based on the two-sided Student’s *t* test while multiple group testing was through one-way analysis of variance (ANOVA) with Tukey multiple comparison test. Statistical significance threshold was set at P<0.05.

## 3 Results

### 3.1 Aged mice show higher lethality after SARS-CoV-2 infection compared to young mice

**Figure 1:**
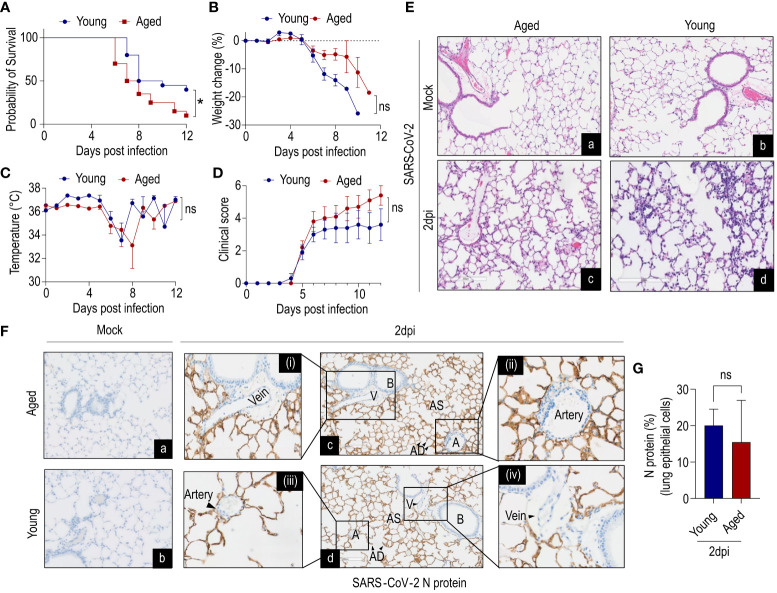
Age-dependent clinical decline of SARS-CoV-2-infected humanized ACE2 mice. K18-hACE2 mice young (12–15 weeks) and aged (85–112 weeks) were inoculated intranasally with 1x10^4^ plaque-forming units (PFU) or received saline (mock). **(A)** Survival, **(B)** %-change in body weight of non-survivors, **(C)** change in body temperature, **(D)** clinical scores were monitored. Aged mice showed less survival compared to young mice, despite no difference in body weight up to 6dpi (data combined from two independent experiments). Data are shown as the mean ± SEM, n=20 mice/group. **(E^a-d^)** Histology of lung cross-sections at 2dpi from K18-hACE2 mice (young and aged) infected with SARS-CoV-2 and mock. n=3 per group; scale bar=200µm. **(F^a-d^)** Immunohistochemistry of N protein in lung cross-sections at 2dpi from K18-hACE2 mice (young and aged) infected with SARS-CoV-2 and mock. n=3 per group; scale bar=200µm. Artery and vein (magnified images) showed no detectable N protein in endothelial cells (ECs). **(G)** Quantification of N protein in lung epithelium (n=3 per group). V=vein; B=bronchiole; AS=alveolar sac; AD=alveolar duct; A=artery. A, Survival of mice was analyzed by the Kaplan–Meier method and the log-rank test. G, Data were analyzed by Student’s t-test. *P<0.05; ns: not significant.

To explore age-related infection outcomes, we investigated the pathogenicity of SARS-CoV-2 in aged (85–112 weeks) and young (12–15 weeks) mice. Due to high lethality rates at 1x10^6^ dose (21, 37), an inoculation dose of 10^4^ PFU of SARS-CoV-2 WA-1 was used for survival experiments. Survival studies revealed a significantly higher mortality rate in aged mice than young mice when challenged with SARS-CoV-2 (**Figure 1A**). Both young and aged mice lost 10% body weight between 8–10dpi and reached a nadir of 25% body weight loss between 10–12dpi (**Figure 1B**). However, no significant differences in body weight were observed. Similarly, there were no significant differences in body temperature between young and aged mice (**Figure 1C**). Although there was no difference in overall clinical score (**Figure 1D**), the aged mice displayed more ruffled fur, hunched postures, and labored breathing compared to young mice at 6dpi.

### 3.2 SARS-CoV-2 infects lung epithelial cells but not ECs in humanized ACE2 mice

We next evaluated the histopathological changes in infected aged and young mice. Histological examination of the lung cross-sections revealed mild-to-moderate multifocal mononuclear immune cell infiltration at 2dpi in both young and aged mice (**Figure 1Ea-d**). In both age groups mononuclear inflammation was observed in peribronchiolar, perivascular, and interstitial compartments, consistent with an interstitial pneumonia. The endothelium was frequently hypertrophied in areas with perivascular mononuclear infiltrates suggestive of dysfunction. Next, we examined viral loads (N protein) in lung cross-sections at 2dpi by immunohistochemistry (**Figure 1F**). Both young and aged mice showed abundant N protein presence in alveolar epithelial cells (type I and II alveolar cells) although there were no significant differences between age groups (**Figure 1G**). Importantly, careful examination of arteries and veins of lung cross-sections revealed the absence of detectable SARS-CoV-2 N protein in ECs (**Figure 1Fc-d**).

In summary, these findings suggest that infection of lung epithelial cells is predominant, while EC infectivity of SARS-CoV-2 is an unlikely event in K18-hACE2 mice at the studied time point.

### 3.3 Transcriptomic profiling of lung ECs from SARS-CoV-2-infected humanized ACE2 mice

**Figure 2:**
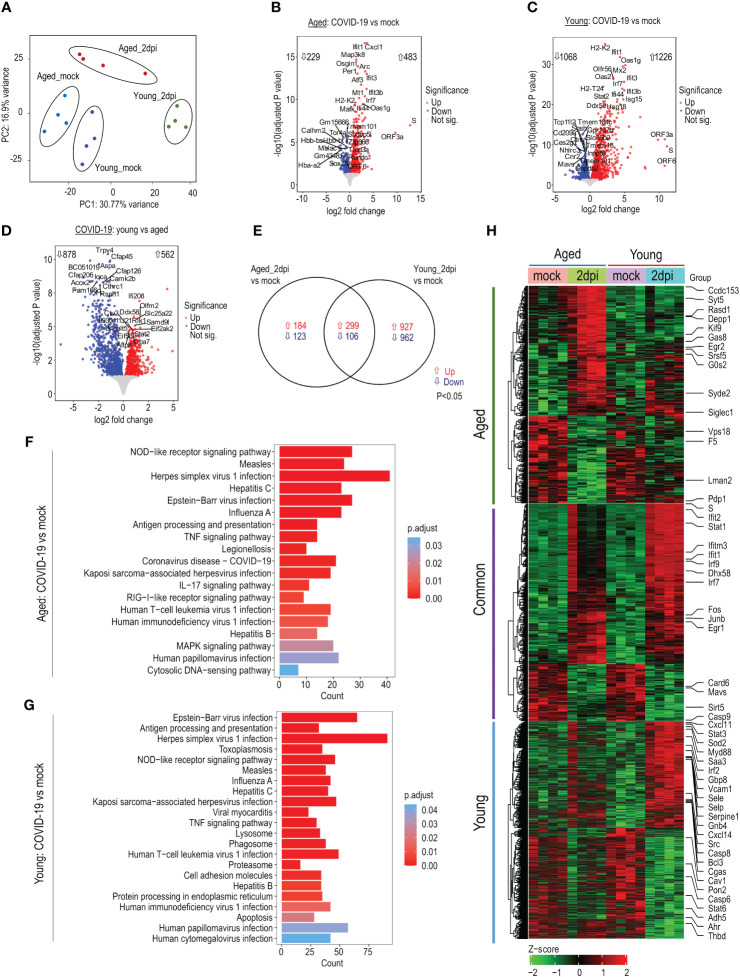
Transcriptomic profile of lung endothelial cells (ECs) during SARS-CoV-2 infection. Young (15 weeks) and aged (72–85 weeks) K18-hACE2 mice were infected with SARS-CoV-2 (1x10^6^ PFU) and lung ECs were isolated at 2dpi. **(A)** Principal component analysis of top 5000 variable genes after regularized log transformation of RNA-seq counts data from lung ECs of young and aged infected and respective mock mice displays age-dependent distinct clusters; n=4 per group. **(B)** Volcano plot of aged_2dpi versus mock. **(C)** Volcano plot of young_2dpi versus mock. **(D)** Volcano plot of young_2dpi versus aged_2dpi. **(E)** Venn diagram of significant differentially expressed genes (adjusted P<0.05) from aged_2dpi and young_2dpi compared to respective mock groups. **(F)** KEGG pathway enrichment analyses of significant (adjusted P<0.05) up- and downregulated differentially expressed genes in aged_2dpi vs mock. **(G)** KEGG pathway enrichment analyses of significant (adjusted P<0.05) up- and downregulated differentially expressed genes in young_2dpi versus mock. **(H)** Heatmap of significant differentially expressed genes (adjusted P<0.05) in aged_2dpi versus mock, young_2dpi versus mock or common to both aged (2dpi) and young (2dpi) infected mice. Normalized expression values (Z-score from -2/green to +2/red). Key genes of interest are labelled.

To investigate the age-dependent alterations during COVID-19, we performed bulk RNA-seq of lung ECs. ECs were MACS isolated and cell purity was evaluated by flow cytometry (**Supplementary Figures 1A–C**). RNA was isolated from highly enriched ECs for bulk RNA-seq. The principal component analysis plot illustrated clear differences between experimental groups (**Figure 2A**). SARS-CoV-2 induced significant changes in the abundance of many genes at 2dpi versus mock in aged mice (n=712; 229↓/483↑; P<0.05; **Figure 2B** **;** **Supplementary Table 1**), young mice (n=2294; 1068↓/1226↑; P*<*0.05; **Figure 2C** **;** **Supplementary Table 2**), and young versus aged mice (n=1440; 878↓/562↑; P<0.05; **Figure 2D** **;** **Supplementary Table 3**). Interestingly, ~83% of the DE genes in young mice (n=1889; P<0.05) and ~43% in aged mice (n=307; P*<*0.05) were specific to each age group (**Figure 2E** **;** **Supplementary Table 4**). A subset of genes was dysregulated in both young and aged mice (n=422; 299↑/106↓; P<0.05; **Figure 2E** **;** **Supplementary Table 4**). Viral transcripts represented <1–2% of all mapped reads at 2dpi.

KEGG enrichment analysis showed predominant upregulation of viral infection pathways (overlapping with Herpes simplex virus 1, influenza A, Measles, and Epstein-Barr virus infection), NOD-like receptor signaling pathway, virus sensing RIG-I-like receptor signaling pathway in both aged and young infected mice (**Figures 2F, G**). IPA enrichment analysis revealed that compared to their respective mock controls, TREM1 signaling, PI3/AKT signaling, and Role of RIG1-like Receptors in Antiviral Innate Immunity pathways were more highly activated in young mice than aged mice (**Supplementary Figure 2A**), while eNOS signaling, and coronavirus pathogenesis pathways were more repressed in aged mice than young mice (**Supplementary Figure 2A**). Similarly, IFN, Death receptor, and Pyroptosis signaling pathways were more activated in infected young mice versus aged mice (**Supplementary Figure 2B**). The heatmap of up- and downregulated genes highlights relevant genes connected to antiviral response, coagulation factors, and apoptosis signaling (**Figure 2H**). In summary, SARS-CoV-2 infection drastically dysregulated the endothelial transcriptome in young and aged mice compared to respective mock controls with young mice showing enhanced responses to infection.

### 3.4 Endothelium from aged mice shows a reduced inflammatory response compared to young mice after infection with SARS-CoV-2

**Figure 3:**
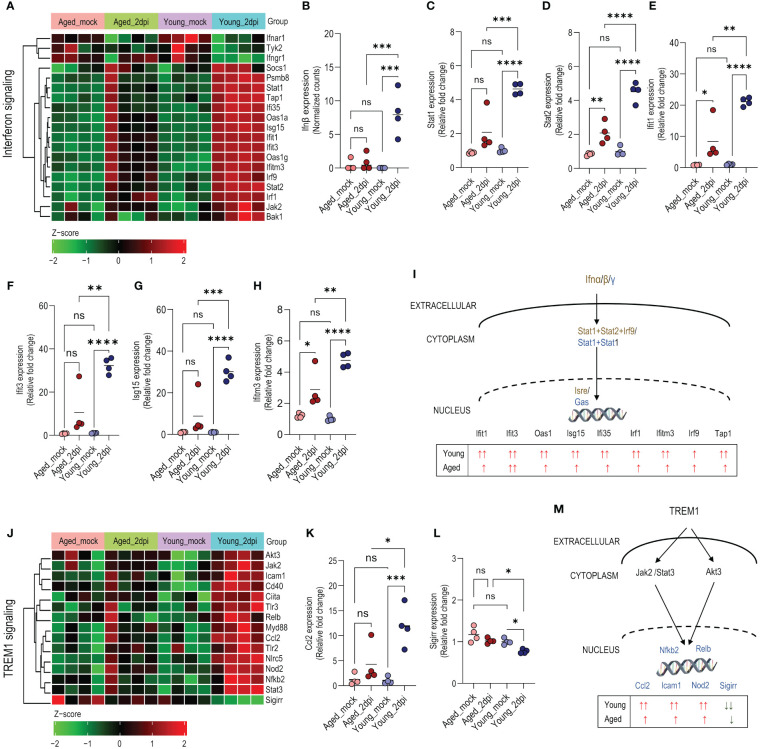
Age-dependent changes in interferon and TREM1 signaling in lung endothelial cells (ECs) during SARS-CoV-2 infection. Ingenuity Pathway Analysis enrichment was performed for significant (adjusted P<0.05) differentially expressed genes in young and aged infected (2dpi) mice versus respective mock controls with tissue filter set to ECs. **(A)** Heatmap of significant differentially expressed genes (adjusted P<0.05) enriched in interferon signaling pathway in young_2dpi versus mock, aged_2dpi versus mock or common to both young (2dpi) and aged (2dpi) infected mice. Normalized expression values (Z-score from -2/green to +2/red). **(B)** *Ifnβ* expression (normalized counts). **(C–H)** Relative expression of genes calculated from normalized count values for the different genes; expression was normalized to young_mock group. **(C)** *Stat1*, **(D)** *Stat2*, **(E)** *Ifit1*, **(F)**, *Ifit3*, **(G)**, *Isg15*, **(H)** *Ifitm3*. **(I)** Schematic diagram of interferon pathway highlighting the distinct expression profile of enriched genes in young and aged mice at 2dpi versus mock. The text in dark gold and blue colors represents elements of the type I and type II interferon response, respectively. **(J)** Heatmap showing enriched genes in the TREM1 signaling pathway based on significant differentially expressed genes (adjusted P<0.05) in young_2dpi versus mock. Corresponding genes from aged_2dpi versus mock are shown for comparison as TREM1 signaling was not significantly enriched in aged (2dpi) infected mice. Normalized expression values (Z-score from -2/green to +2/red). **(K, L)** Relative expression of genes calculated from normalized count values for the different genes; expression was normalized to young_mock group. **(K)** *Ccl2*, **(L)** *Sigirr.* **(M)** Schematic diagram of TREM1 pathway highlighting the distinct expression profile of enriched genes in young and aged mice at 2dpi versus mock. **(B–H, K, L)**: Data are shown as the mean, n=4 mice/group; data were analyzed through one-way ANOVA followed by Tukey multiple comparison test. *P<0.05; **P<0.01; ***P<0.001; ****P<0.0001; ns: not significant.

Aging is accompanied by progressive biological changes in the immune system leading to a functional decline as evidenced by increased susceptibility to respiratory infections such as influenza and novel coronaviruses (38). Thus, we investigated changes in IFN signaling in lung ECs from young and aged mice at 2dpi. Young mice displayed a more pronounced IFN response compared to aged mice (**Figure 3A**). For instance, IFNβ (while only weakly expressed at the studied 2dpi time point) was upregulated in young mice compared to mock controls and aged mice, while aged mice demonstrated no difference compared with mock controls (**Figure 3B**). *Stat1* (type I, II, III) and *Stat2* (type I, III) are key transcription factors of IFN signaling, and are essential components of the cellular antiviral response and adaptive immunity (39). *Stat1* and *Stat2* expressions were ~2-fold higher in young mice compared to aged mice (**Figures 3C, D**). The IFIT gene family encodes defense proteins that are induced after viral infection or pathogen-associated molecular pattern recognition (40). ECs from young mice expressed ~20-fold higher *Ifit1*, *Ifit3*, and *Isg15* compared to mock controls; aged murine ECs exhibited relatively less induction of *Ifit1, Ifit3*, and *Isg15* than young mice (**Figures 3E–G**). Similarly, *Ifitm3* induction was lower in old mice (~2-fold) during infection relative to young mice (~5-fold) (**Figure 3H**). A schematic diagram of the signaling pathways based on the expression profile in young and aged mice at 2dpi is presented in **Figure 3I**.

Pathway analysis revealed that Triggering Receptor Expressed on Myeloid cells-1 (TREM1) signaling was highly activated in young mice compared to aged mice (**Supplementary Figure 2A**). TREM1 is an immunoreceptor expressed on neutrophils, monocytes/macrophages, and ECs (41). It amplifies the inflammatory response driven by Toll-Like Receptor (TLR) engagement. Several TREM1-associated signaling molecules, receptors, and transcriptional factors (*Tlr2*, *Tlr3*, *Cd40*, *Myd88*, *Jak2*, *Stat3*, *Akt3, Icam1*) were significantly more upregulated in young mice than in aged mice (**Figure 3J**). Induction of TREM1-mediated chemokine ligand 2, *Ccl2*/MCP1 (42), was profoundly abrogated in aged mice relative to young mice (**Figure 3K**). Interestingly, the negative regulator, *Sigirr* (single immunoglobulin IL-1R-related molecule) (43), was significantly downregulated in young mice with comparatively less downregulation in aged mice versus the respective mock controls (**Figure 3L**). The schematic diagram shows the signaling pathways based on the expression profile in young and aged mice at 2dpi (**Figure 3M**).

The pathway analyses above highlight key inflammatory responses that are activated by the regulated genes in response to infection. However, biological responses are typically not driven by a single gene but a set of genes that are regulated together and share similar expression patterns. To identify whether coregulated genes in aged and young mice define specific responses during infection with SARS-CoV-2, we performed WGCNA (33, 35). In total, we identified 87 modules of co-expressed genes (module eigengenes). The correlation between the modules and experimental groups is presented in **Supplementary Figure 3**. Infection status was significantly correlated with module ME1 (**Supplementary Figure 3** **).** Genes in ME1 were highly induced in young mice 2dpi, while aged 2dpi mice showed diminished induction (**Supplementary Figures 4A, B**). Genes positively correlated with ME1 were enriched in host defense processes such as interferon response and cytokine production (**Supplementary Figure 4C**). ME1 genes included chemokines (e.g., *Cxcl9*, *Cxcl10*, *Cxcl11*), interferon-induced genes (*Isg15*, *Rsad2*) and genes involved in viral sensing (*Oasl1*) that were highly induced in young mice 2dpi compared to young mock controls. ME1 genes were also induced in infected aged mice, albeit at a lower level than infected young mice, and included immune-related genes such as *Ifit1*, *Rsad2* and *Oasl1* (**Supplementary Table 5**). The complete gene-module and gene-group correlation data is presented in **Supplementary Table 6**.

Overall, these findings suggest that senescent lung ECs mount an impaired immune response at 2dpi in humanized ACE2 mice, suggesting delayed pathogen defense mechanisms that might contribute to delayed viral clearance and reactive hyperinflammation, which in turn lead to high mortality.

### 3.5 Endothelium from aged mice displays distinct cytokine/chemokine and acute phase response signaling than young mice infected with SARS-CoV-2

**Figure 4:**
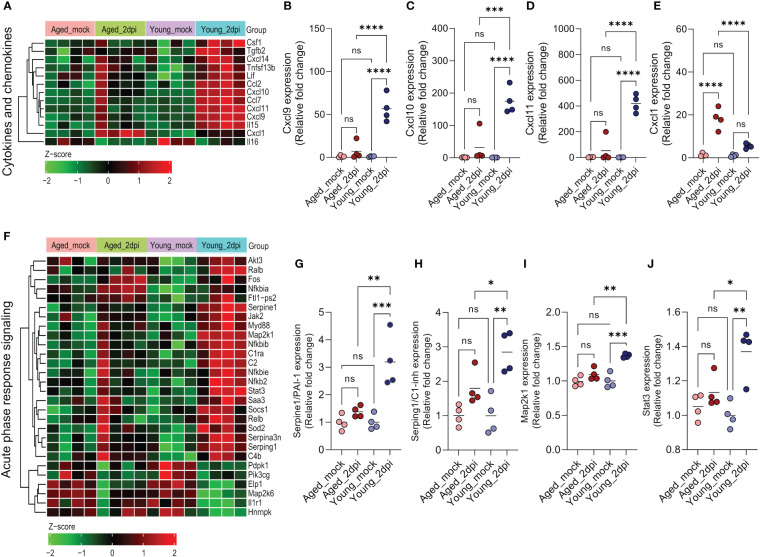
Age-dependent changes in cytokines/chemokines and acute phase response signaling in lung endothelial cells (ECs) during SARS-CoV-2 infection. Ingenuity Pathway Analysis enrichment was performed for significant (adjusted P<0.05) differentially expressed genes in aged and young infected (2dpi) mice versus respective mock controls with tissue filter set to ECs. **(A)** Heatmap of significant differentially expressed cytokines and chemokines (adjusted P<0.05) in aged_2dpi versus mock, young_2dpi versus mock or common to both aged (2dpi) and young (2dpi) infected mice. Normalized expression values (Z-score from -2/green to +2/red). **(B–E)** Relative expression of genes calculated from normalized count values for the different genes; expression was normalized to young_mock group. **(B)** *Cxcl9*, **(C)** *Cxcl10*, **(D)** *Cxcl11*, **(E)** *Cxcl1*. **(F)** Heatmap of acute phase response signaling associated significant differentially expressed genes (adjusted P<0.05) in aged_2dpi versus mock, young_2dpi versus mock or common to both aged (2dpi) and young (2dpi) infected mice. Normalized expression values (Z-score from -2/green to +2/red). **(G–J)** Relative expression of genes calculated from normalized count values for the different genes; expression was normalized to young_mock group. **(G)** *Serpine1*/PAI-1, **(H)** *Serping1*/C1-inh, **(I)** *Map2k1*, **(J)** *Stat3*. **(B–E, G–J)** Data are shown as the mean, n=4 mice/group; data were analyzed through one-way ANOVA followed by Tukey multiple comparison test. *P<0.05; **P<0.01; ***P<0.001; ****P<0.0001; ns: not significant.

As the aged mice demonstrated defective IFN response (**Figure 3A**), we examined age-dependent expression changes in cytokine, chemokine, and acute phase response signaling in lung ECs during SARS-CoV-2 infection at 2dpi. Young mice exhibited a more prominent cytokine/chemokine response in lung ECs than aged mice (**Figure 4A**). Consistent with the IFN response genes, IFN-regulated chemokines (44), such as *Ccl2* (~10-fold), *Cxcl9* (~50-fold), *Cxcl10* (~180-fold), and *Cxcl11* (~400-fold), were significantly upregulated in young mice compared to aged mice (**Figures 3K** and **4B–D**). Notably, aged mice ECs manifested significantly higher *Cxcl1* expression (~20-fold) compared with young mice (**Figure 4E**).

Acute-phase response genes are directly activated as a part of the adaptive stress response during infection and various other disease states. Acute-phase protein concentrations are altered with disease severity in COVID-19 patients, suggesting their potential utility in diagnosis and treatment (45). In our study, lung ECs after SARS-CoV-2 infection of young mice showed an increased activation of acute-phase response signaling in comparison to aged mice (**Figure 4F**). Various transcriptional factors were significantly upregulated (e.g., *Ralb*, *Relb*, and *Nfkb2*) or downregulated (e.g., *Elp1*, *Map2k6*) in young mice relative to old mice. Complement genes like *C1ra*, *C2* and *C4b* were especially highly induced in young mice. Aging was associated with a diminished capacity to induce acute-phase genes like PAI-I (*Serpine1*) and C1-inh (*Serping1*) as well as transcriptional factors such as *Map2k1* and *Stat3* (**Figures 4G–J**).

In conclusion, aged lung ECs exhibited impaired cytokine/chemokine expression and inadequate acute phase response signaling in humanized ACE2 mice at 2dpi.

## 4 Discussion

The susceptibility to SARS-CoV-2 infection increases proportionally with age, placing older individuals at a significantly higher risk of developing severe COVID-19. Therefore, gaining insight into age-dependent pathological changes during SARS-CoV-2 infection is imperative for effectively safeguarding vulnerable populations. To better characterize the contribution of lung ECs to the age-dependent pathology of COVID-19, including endothelial infectivity, we performed a transcriptomic study in K18-hACE2 mice infected with SARS-CoV-2. Our unbiased bulk RNA-seq approach demonstrated that lung ECs in aged mice displayed an impaired inflammatory response compared to young mice. Young mice mounted several-fold higher IFN responses (*Ifitm3, Ifit1, Ifit3, Isg15*) and IFN-induced chemokines (*Cxcl10* and *Cxcl11*) than aged mice, indicating a markedly dampened immune response with aging. Furthermore, lung epithelial cell infectivity was predominant, and endothelial infectivity of SARS-CoV-2 seems an unlikely event at 2dpi in humanized ACE2 mice.

We noticed that aged K18-hACE2 mice infected with SARS-CoV-2 showed significantly higher mortality compared to young mice, in line with previous studies (46, 47). Both young and aged mice exhibited increased inflammation in peribronchiolar, perivascular, and interstitial compartments, consistent with interstitial pneumonia in both age groups. Immunohistochemistry analysis provided additional insights, revealing a significant presence of the N protein in alveolar epithelial cells in both young and aged mice. Quantification of the N protein showed no significant difference between the two age groups at 2dpi, suggesting that the viral load in the lung is independent of age and alternative factors may contribute to the higher lethality in aged mice. This finding contrasts with the higher viral burden in older mice especially at 4dpi reported by Chen et al., although their study involved the B.1.1.7 variant and quantified viral loads based on SARS-CoV-2 RNA-dependent RNA polymerase, which may account for the differences (46).

The vascular endothelium, as the innermost layer of blood vessels, functions as a dynamic interface between circulating blood and various tissues and organs, playing a crucial role in maintaining tissue homeostasis (48). SARS-CoV-2 infection of endothelium is less studied than airway epithelium and alveolar pneumocytes. Accumulating evidence suggests that COVID-19 affects the pan-vasculature in the extrapulmonary systems by directly (via virus infection) or indirectly (via cytokine storm) (49–51) causing endothelial dysfunction (endotheliitis, endothelialitis and endotheliopathy) and multi-organ injury (52). The elevated D-dimer and thrombocytopenia in severe COVID-19 cases may be attributed to dysregulated inflammation and the formation of microthrombi, complicated by endothelial dysfunction (53). This underscores the importance of aggressively addressing endothelial impairment to mitigate thrombotic events. Despite the thrombo-inflammatory phenotype, direct SARS-CoV-2 infection of ECs or the presence of viral particles has not been definitively demonstrated in existing animal models or human biopsies (54). According to early observations, SARS-CoV-2 infects ECs and induces vascular complications (11, 55). However, owing to the challenges of interpreting transmission election microscopy and the high observer variability of those images, the presence of viral particles in the endothelium remains debatable (56, 57).

Transcriptomic analysis of lung ECs from young and old K18-hACE2 mice infected with SARS-CoV-2 at 2dpi unraveled age-dependent endothelial signatures of COVID-19. Despite SARS-CoV-2 infection drastically dysregulating the lung endothelial transcriptome in both groups compared to respective mock controls, the proportion of DEGs was relatively higher in young mice than aged mice. Pathway analysis revealed that SARS-CoV-2 infected young mice exhibited an amplified endothelial IFN response, significantly attenuated in aged mice. In support of our findings, Chen et al. demonstrated earlier using homogenized lung tissues that the innate IFN response and adaptive antibody response against SARS-CoV-2 infection were significantly impaired in aged mice compared to young mice (46). Thus, a diminished IFN response may curtail anti-viral response against SARS-CoV-2 infection in lung ECs of aged mice compared to young mice. Additionally, the low autochthonous IFN expression in the ECs of our study supports endothelial dysfunction in COVID-19 being mediated indirectly due to proximity to inflamed IFN-producing epithelium. In our datasets, TREM1 signaling and cytokines were elevated to a greater extent in young mice than aged mice, which is in contrast to clinical studies (58). Overall, the endothelium of aged mice at 2dpi showed a dampened inflammatory profile relative to young mice. These differences could potentially be explained by the early infection time point of our experiments, which may correspond to the presymptomatic phase when compared to data from human patients, during which blood samples and lung autopsy samples are typically not available and are sampled at later time points. An insufficiently low inflammatory response in the ECs of older mice during the early stage could allow for the spread of infection and, consequently, lead to reactive hyperinflammation and lower survival.

The inflammatory response is an essential host defense mechanism. However, exacerbated inflammatory responses can be catastrophic leading to tissue injury, multi-organ damage and mortality, as observed in sepsis. On the flip side, age-associated blunting of the interferon response may also be catastrophic due to diminished ability to fight against the infection. While tissue/cell type, type of pathogen, and the stage of infection dictate the magnitude and type of immune response, certain patterns of host response are common across pathogens and tissues. For instance, in a study by Lee et al. (59) on peripheral blood mononuclear cells, both patients with severe COVID-19 and influenza showed elevated expression of immune-related genes such as *TLR2*, *IFI35*, and *NFKBIA* compared to the respective healthy controls (59). Another study by Wang et al. on whole lung transcriptome observed an increased induction of pro-inflammatory genes such as *Saa3*, *Cxcl10*, and *Cxcl11* in both influenza and COVID-19 infected mice (60).

Infection and inflammation trigger the induction of acute-phase reaction proteins as part of the host stress response. Acute-phase reaction proteins, produced primarily by hepatocytes with contribution from other cells such as ECs, have been implicated in pathogenesis, disease severity, mortality, and even post-acute sequelae of COVID-19 (45, 61). In line with the curtailed inflammatory response in aged mice during COVID-19, the acute phase proteins were relatively less abundant in the aged endothelium compared to young mice, which demonstrated a hyperactive response consistent with clinical reports.

Finally, it is possible that severe disease and pathology are driven by local lung infection in epithelial cells which leads to release of mediators triggering endothelial dysfunction. In conclusion, our study suggests that a suppressed immune landscape is a key driver of age-associated endothelial dysfunction during COVID-19. Targeting these immune pathways in ECs may have prognostic and therapeutic benefits although further studies, including dissecting these functional changes at a single-cell level, are needed.

## Data availability statement

The datasets presented in this study can be found in the NCBI Gene Expression Omnibus (GEO) repository under the accession number: GSE260604.

## Ethics statement

The animal study was approved by Boston University Biomedical Research, Institutional Biosafety Committee and Institutional Animal Care and Use Committee (IACUC). The study was conducted in accordance with the local legislation and institutional requirements.

## Author contributions

SS: Conceptualization, Formal analysis, Funding acquisition, Investigation, Methodology, Project administration, Resources, Supervision, Visualization, Writing – original draft, Writing – review & editing. DK: Investigation, Writing – review & editing, Visualization. AJ: Data curation, Formal analysis, Visualization, Writing – review & editing. AO’C: Investigation, Writing – review & editing. SW: Formal analysis, Investigation, Visualization, Writing – review & editing. PM: Investigation, Writing – review & editing. CR: Writing – review & editing. GC: Writing – review & editing. NC: Funding acquisition, Investigation, Supervision, Writing – review & editing. FD: Funding acquisition, Supervision, Writing – review & editing. MB: Conceptualization, Funding acquisition, Resources, Supervision, Writing – review & editing.
