# Supplementary material for: Aging is associated with an insufficient early inflammatory response of lung endothelial cells in SARS-CoV-2 infection
Source: Front Immunol. 2024 Jun 7;15:1397990. doi: 10.3389/fimmu.2024.1397990 (PMC11190167; doi:10.3389/fimmu.2024.1397990)
Supplement: Supplementary file 7 [file Image_1.pdf]

Supplementary Material

Aging is Associated with an Insufficient Early Inflammatory Response of Lung Endothelial Cells in SARS-CoV-2 Infection

Saravanan Subramaniam[1], Devin Kenney[2,3], Archana Jayaraman[1], Aoife Kateri O'Connell[2,4], Sarah Walachowski[1,5], Paige Montanaro[3,4], Christoph Reinhardt[5,6], Giuseppe Colucci[7,8], Nicholas A Crossland[2,3,4], Florian Douam[2,3], Markus Bosmann[1,2,5,*]

*Corresponding author: Dr. Markus Bosmann: mbosmann@bu.edu

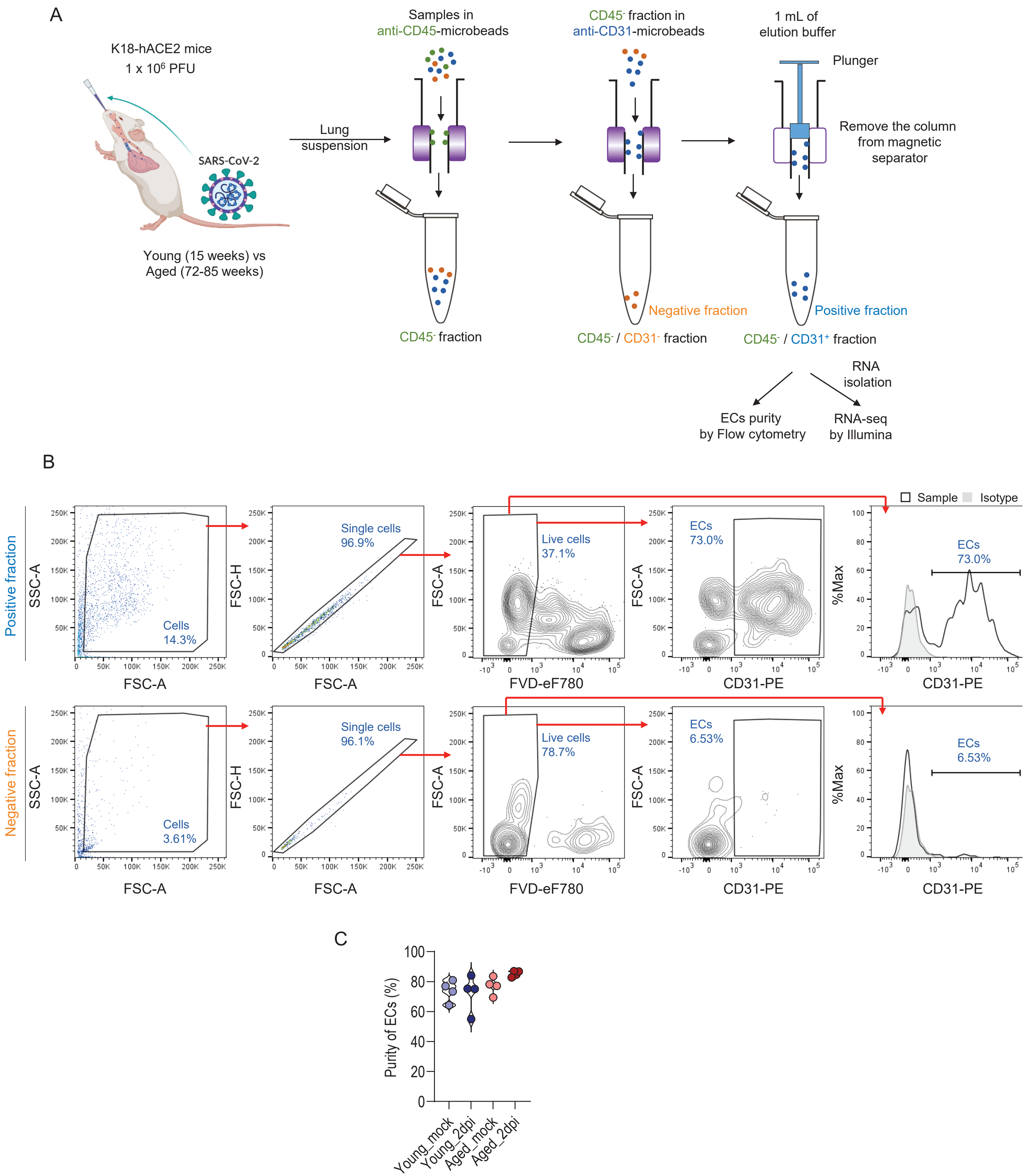

Supplementary Figure 1. Isolation of endothelial cells (ECs). (A) K18-hACE2 mice were inoculated intranasally with $1x10^6$ plaque-forming units (PFU) or received saline (mock). After 2dpi, whole lungs were digested, and ECs were isolated using a two-step column-based magnetic microbead separation protocol for CD45 and CD31. The purity of ECs (collected in the positive fraction eluted from the second step of selection, i.e. $CD45^-CD31^+$ cells) was assessed by flow cytometry and remaining samples were used for RNA-seq analysis. (B) Flow cytometry gating strategy of single, viable (FVD^-), $CD45^-$/$CD31^+$ lung ECs (positive fraction) and residual ECs in the $CD45^-$/$CD31^-$ negative fraction. (C) Relative purity of ECs isolated at 2dpi from lungs of K18-hACE2 mice from different groups.

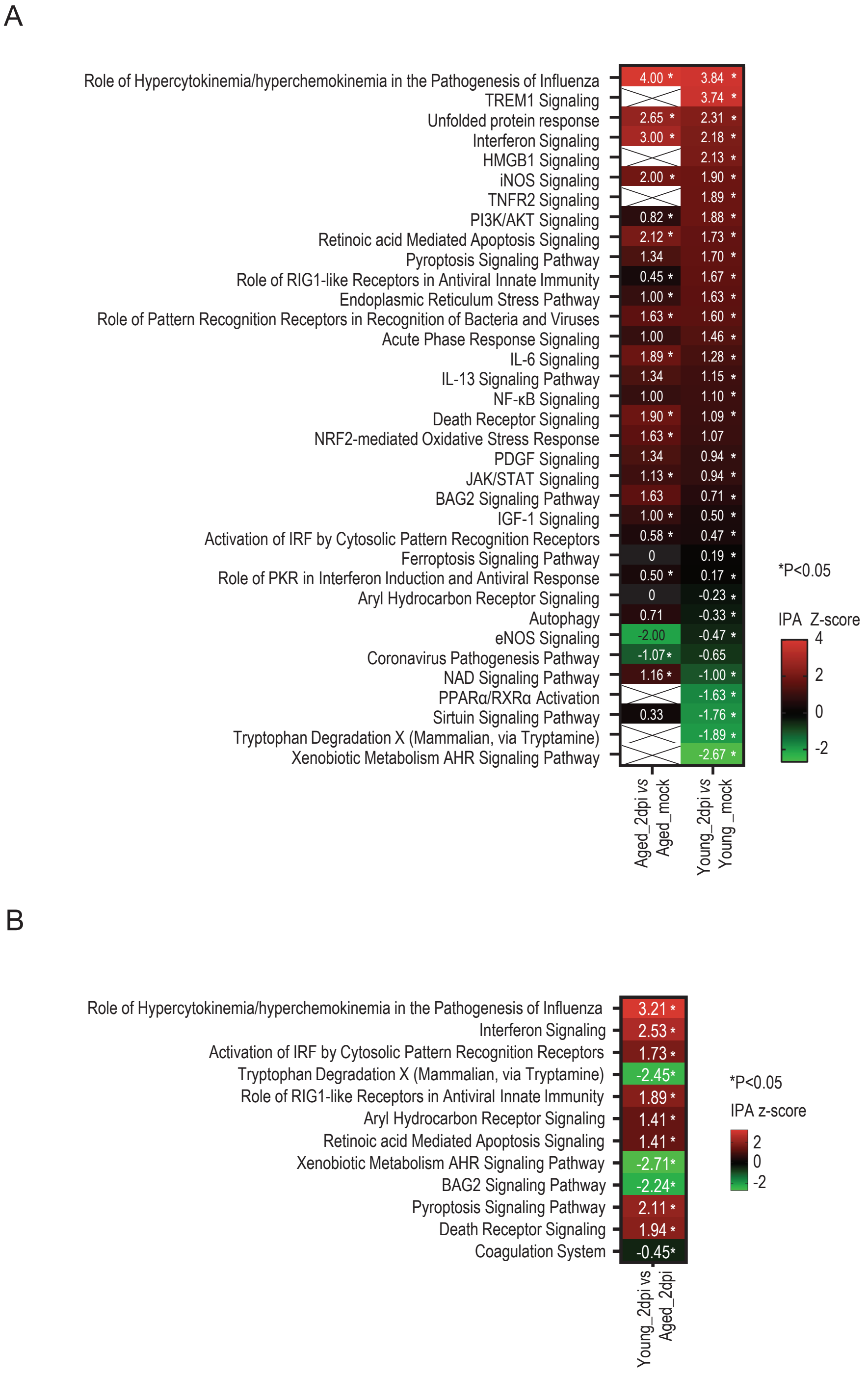

Supplementary Figure 2. Key pathways enriched in young and aged infected mice based on Ingenuity Pathway Analysis (IPA). (A) IPA enrichment was performed for significant (adjusted P<0.05) differentially expressed genes in aged_2dpi versus mock and young_2dpi versus mock with tissue filter set to endothelial cells (ECs). The comparison analysis was performed on the significant Fisher's test P value <0.05 enrichment results from young and aged mice data. All non-relevant terms were filtered. (B) IPA enrichment was performed for significant (adjusted P<0.05) differentially expressed genes in young_2dpi versus aged_2dpi with tissue filter set to ECs. All non-relevant terms were filtered out from the significant pathways (Fisher's test P<0.05).

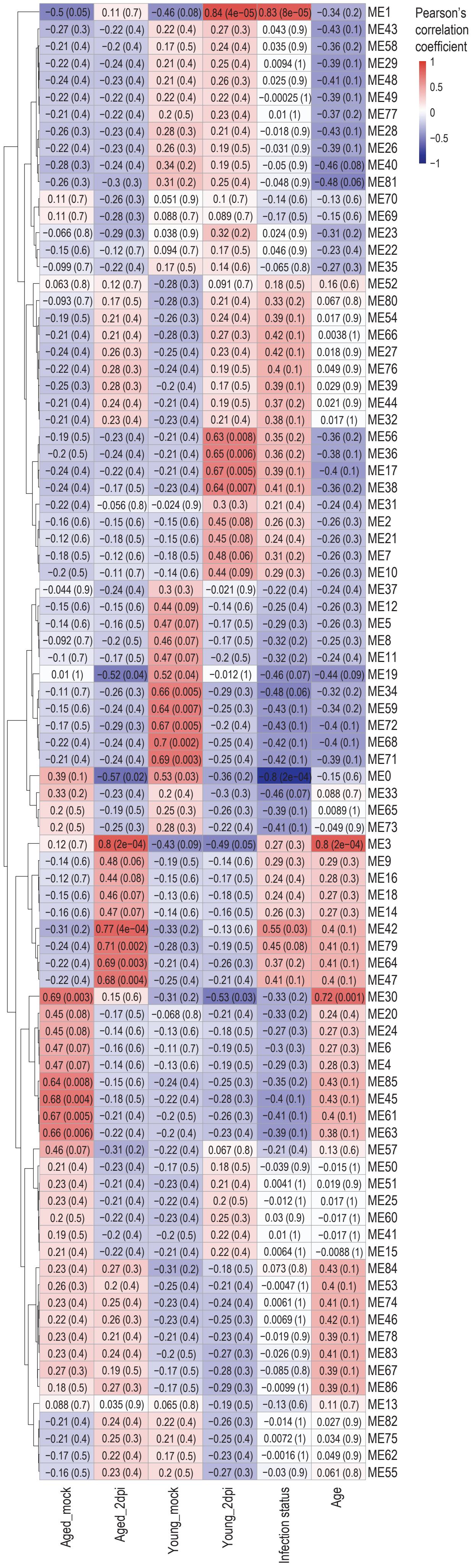

Supplementary Figure 3. Heatmap of module-group correlation. Correlation was estimated between the module eigengenes and experimental groups. Each row represents a module and columns correspond to experimental groups including aged mock, aged 2dpi, young mock, young 2dpi, infection status (infected or uninfected), and age (aged or young). The values in each cell represents Pearson's correlation coefficient with the student asymptotic p-values included within brackets.

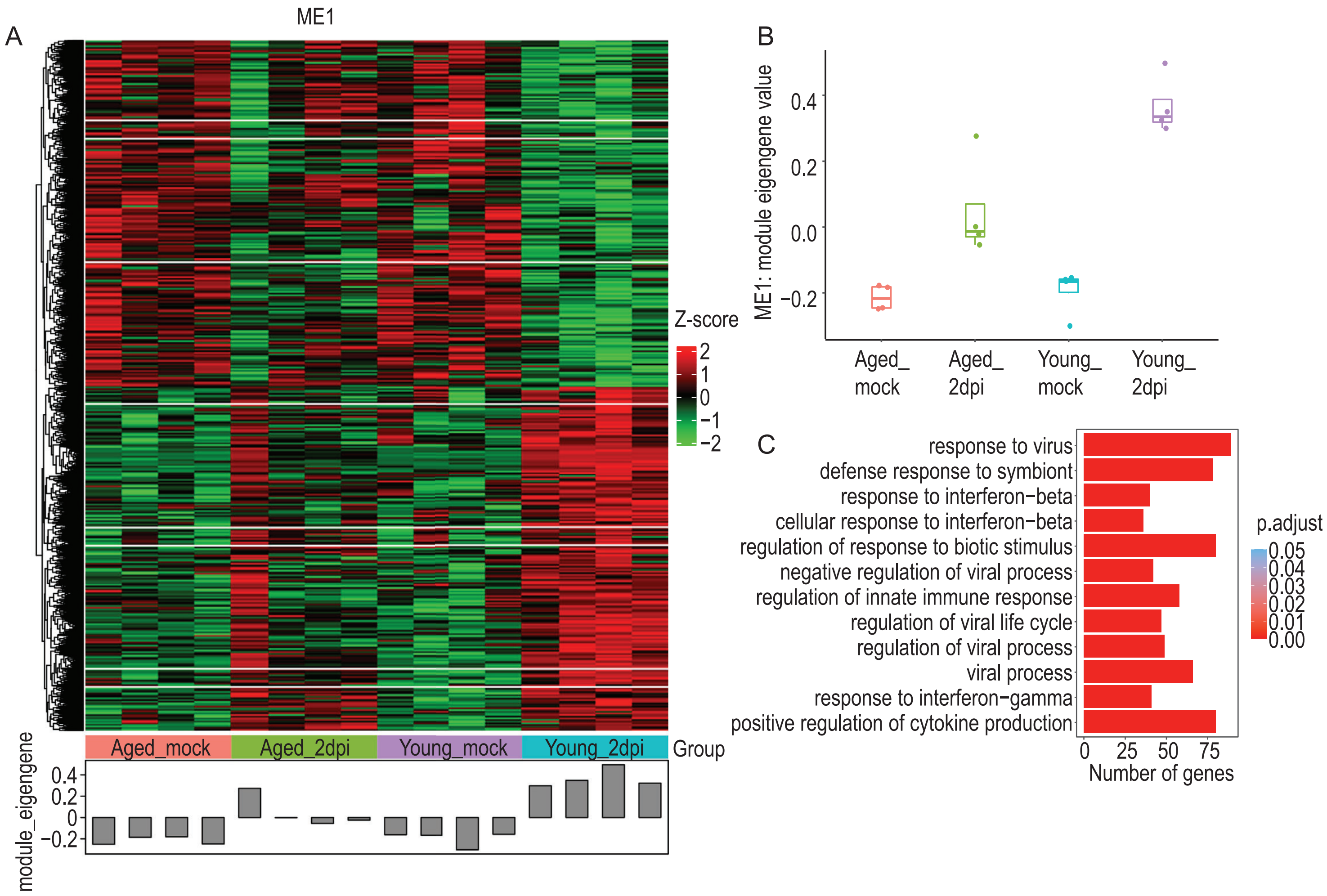

Supplementary Figure 4. Module ME1 is highly correlated with SARS-CoV-2 infection in aged and young mice. (A) Heatmap of co-expressed genes in module ME1 (n=4380 genes) shows greater induction in endothelial cells of young 2dpi mice compared with aged 2dpi mice. Rows of the heatmap represent genes and columns represent samples. Normalized expression values (Z-score from -2/green to +2/red). The bar plot represents the eigengene value for module ME1 for each mouse. (B) Boxplot showing the increased expression of ME1 module genes in infected young mice compared to all other groups. Infected aged mice exhibited dampened induction of ME1 genes compared with young mice. (C) Bar plot of relevant gene ontology biological processes that were enriched among genes from module ME1. Enrichment analysis was based on genes that were significantly, positively correlated with ME1.

Supplementary Tables

Supplementary Table 1. Normalized counts and differential expression from bulk RNA-seq of lung endothelial cells of aged (72-85 weeks) SARS-CoV-2 infected mice compared with mock mice.

Supplementary Table 2. Normalized counts and differential expression from bulk RNA-seq of lung endothelial cells of young (15 weeks) SARS-CoV-2 infected mice compared with mock mice.

Supplementary Table 3. Normalized counts and differential expression from bulk RNA-seq of lung endothelial cells of young (15 weeks) SARS-CoV-2 infected mice compared with aged (72-85 weeks) SARS-CoV-2 infected mice.

Supplementary Table 4. List of statistically significant differentially expressed genes that are up- and downregulated specifically in the aged SARS-CoV-2 infected mice or young SARS-CoV-2 infected mice or both age groups compared with respective mock mice.

Supplementary Table 5. Normalized counts of genes with significant positive correlation (correlation >0, correlation P<0.05) with weighted gene co-expression network analysis module ME1 in aged and young SARS-CoV-2 infected and mock mice.

Supplementary Table 6. Data from weighted gene co-expression network analysis shows correlation between the module eigengene and gene expression, and gene expression and experimental groups.
